# Modified Dioscorea pills ameliorate cognitive impairment induced by chronic cerebral hypoperfusion via restoring the hippocampal neurovascular unit

**DOI:** 10.3389/fmed.2026.1921297

**Published:** 2026-10-20

**Authors:** Qian Xu, Dan He, Hongbing Li

**Affiliations:** 1 Neurology Department, Ezhou Central Hospital, Ezhou, Hubei, China; 2 Emergency Department, First Hospital of Guiyang, Guiyang, Guizhou, China

**Keywords:** chronic cerebral hypoperfusion, cognitive impairment, hippocampus, modified Dioscorea pills, neurovascular unit, vascular dementia

## Abstract

**Objective:**

Chronic cerebral hypoperfusion (CCH) is a major cause of progressive cognitive decline and vascular dementia in older adults. Modified Dioscorea Pills (MDP), a traditional Chinese medicinal formula, has demonstrated clinical efficacy for dementia treatment. However, the effects and molecular mechanisms of MDP on the hippocampal neurovascular unit (NVU) in CCH remain unclear. This study aimed to explore whether MDP alleviates CCH-induced cognitive impairment by protecting and restoring the hippocampal NVU in rats.

**Methods:**

A rat CCH model was established via bilateral common carotid artery occlusion (BCCAO). Forty SPF-grade male Sprague-Dawley rats were randomly divided into Sham, CCH model and MDP treatment groups. Rats received intragastric administration of normal saline or MDP concentrated decoction for 45 consecutive days. The Morris Water Maze test was used to assess learning and memory functions. Immunohistochemistry was applied to detect the expression of apoptosis-, inflammation-, neuron-, synapse-, glia-, myelin- and endothelial-related proteins, as well as microvessel density (MVD). Western blot was used to quantify Ang-1, Ang-2, Tie2 and VEGF levels. Transmission electron microscopy (TEM) was performed to observe neuronal and synaptic ultrastructure. Pearson correlation analysis was used to evaluate the correlations among NVU-related indicators.

**Results:**

MDP significantly improved learning and memory deficits in CCH rats. MDP downregulated NF-*κ*B p65 expression and the Bax/Bcl-2 ratio, consistent with the attenuation of neuroinflammation and apoptosis-associated signaling, inhibited excessive gliosis, and upregulated neuronal, synaptic and myelin-related protein levels. MDP further activated the Ang/Tie2/VEGF pathway and reduced the abnormally elevated CD34 expression and microvessel density (MVD), indicating that MDP promoted vascular stabilization and remodeling of the excessive immature microvasculature rather than further pathological angiogenesis. TEM confirmed that MDP relieved neuronal nuclear damage and synaptic destruction. Correlation analysis revealed statistically significant associations among multiple components of the hippocampal NVU, whereas inflammation-related markers were negatively associated with NVU integrity.

**Conclusions:**

MDP ameliorates CCH-induced cognitive impairment by comprehensively restoring the structure and function of the hippocampal NVU. The mechanisms involve attenuation of neuroinflammation and apoptosis-associated signaling, protection of neurons and synapses, inhibition of pathological gliosis, restoration of oligodendrocyte-lineage integrity, and stabilization and remodeling of the hippocampal microvasculature. This work provides preclinical evidence for the clinical application of MDP in CCH and vascular dementia.

## 1 Introduction

Chronic cerebral hypoperfusion (CCH) is a core pathological feature of cerebral small vessel disease. In the early stage of CCH, the brain may upregulate the oxygen extraction fraction to compensate for hypoxia and mitigate sustained damage. But persistent and severe hypoperfusion eventually causes white matter injuries (WMI), cortical damage-associated neurological dysfunction impairments (1), and multi - domain cognitive damage, which progresses to to vascular dementia (VD) in the advanced stage. Metabolically, CCH induces cerebral energy imbalance, causing mitochondrial damage, excessive reactive oxygen species (ROS), and triggering neuronal apoptosis through endoplasmic reticulum stress and intracellular calcium overload. CCH also activates astrocytes and microglia via damage - associated molecular patterns (DAMP), thereby initiating a robust neuroinflammation. Moreover, these mechanisms further induce axon degradation, synaptic dysfunction, and impair nitric oxide (NO) production to disrupt cerebral blood flow, which exacerbates secondary cerebral damage (2, 3).

CCH severely impairs the physical and mental health of the elderly. With the aging population and increasing prevalence of carotid atherosclerosis, CCH is highly common in the elderly individuals and serves as a key pathologic driver for the onset and progression of VD (4). Most patients with CCH present with irreversible cerebral hypoperfusion status that is difficult to correct (5), and current medications—whether monotherapy or combination therapy, yield unsatisfactory therapeutic effects for CCH (6). Notably, recent high-impact clinical and translational evidence demonstrates that carotid revascularization such as carotid endarterectomy (CEA), can ameliorate cognitive impairment and restore cerebral blood flow and white matter integrity in patients with carotid artery stenosis-associated CCH, with upregulation of Nrf2 antioxidant signaling and suppression of neuroinflammation as core mechanisms. Untreated CCH often progresses to severe dementia and its associated complications, ultimately leading to poor clinical outcomes and even death. Recently neurovascular unit (NVU) research shows that neurons release cytokines to regulate cerebral microcirculation, perfusion, and blood - brain - barrier permeability (7). Vascular endothelium is also involved in the regulation of neuronal plasticity (8). Furthermore, NVU participates in protease homeostasis, neuro - immune interacting, metabolic waste clearance, brain temperature regulation, and experience - dependent hippocampal neurogenesis (9). NVU research has deepened our understanding of the intercellular signaling mechanisms between neurons, glial cells, and local microvessels. and highlighted the highly coordinated response of these components to pathological damage for the maintenance of normal neural tissue structure and cerebral function homeostasis.

Modified Dioscorea Pills (MDP), a traditional Chinese medicine prescription composed of 14 herbs including *Dioscorea opposita, Rehmannia glutinosa, Eucommia ulmoides, polygonum multiflorum, Polygala japonica, and Poria*, has been clinically used for dementia treatment. Clinical studies have shown that MDP can significantly increase mini-mental state examination (MMSE) scores and decrease syndromes differentiation scale for dementia (SDSD), activities of daily living (ADL) scores in VD and Alzheimer's disease (AD). Its potential therapeutic mechanism may involve the promotion of neural nerve repair (10, 11), our former studies also show that MDP promotes neuroplasticity and vascular remodeling effects in CCH (12, 13), but the specific effects of MDP on the constituent components of the hippocampal NVU in CCH remain largely unexplored. In this study, we established a rat CCH model via BCCAO to investigate the interventional effects of MDP on hippocampal NVU damage and the underlying mechanisms, aiming to provide experimental evidence for the clinical application of MDP in CCH and VD treatment.

## 2 Materials and methods

### 2.1 Experimental Animals

40 male specific pathogen free (SPF) Sprague-Dawley (SD) rats, aged 5–6 weeks with a body weight of 180–220 g, were purchased from the Hubei Provincial Research Center for Laboratory Animals [License No. SCXK (E) 2020 - 0018]. They were housed at the Laboratory Animal Center of Guizhou Medical University under a 12 - h light/dark cycle, temperature (22 ± 2) °C, and humidity (60 ± 10)%. After a 7 - day acclimatization period, the experiment began. All procedures, including modeling and tissue collection, were complied with the IACUC guidelines and were approved by the Ethics Committee of Guizhou Medical University (No. 2100819).

### 2.2 Experimental protocols

#### 2.2.1 Animal grouping and intervention

After acclimatization, rats were divided into a CCH group (30 rats) and a sham group (10 rats). Randomization of animals into groups was performed using a random number table. CCH was induced using the modified BCCAO method. Under anesthesia by 4% isoflurane inhalation, the right common carotid artery (CCA) was isolated, ligated at two points apart 0.5 cm, severed between ligated points, then the skin sutured, followed by a local injection of 400,000 units of penicillin. The left carotid artery was treated similarly a week later. In the sham group, CCAs were isolated but not ligated or severed.

After operation, 1 rat in the sham group and 5 rats in the CCH group died. Gavage began one week after BCCAO. The 25 surviving CCH rats were randomized into a MDP group (13 rats) and a model group (12 rats). The model and sham groups received equal volumes of saline using the same method (Figure 1). Final group sizes were 9 (Sham), 12 (Model), and 13 (MDP) rats. No significant differences in body weight or general condition were observed among the three groups before the intervention, confirming that post-operative mortality did not introduce baseline imbalances across groups. All behavioral assessments and histological, immunohistochemical, and Western blotting analyses were conducted by investigators blinded to group allocation. The MDP group received MDP concentrated decoction (1 mL/100 g body weight, once daily, equivalent to 10 mL/kg) for 45 days. Given that 1 mL of the decoction contains approximately 1 g of concentrated extract derived from the raw herbs, the actual administered dose was approximately 10 g extract·kg⁻^1^·d⁻^1^. Using the body surface area-based conversion coefficient of 0.162 (rat-to-human), this corresponds to a human equivalent dose of approximately 1.62 g·kg⁻^1^·d⁻^1^, which is in line with the clinically used dose range of the formula (14).

**Figure 1:**
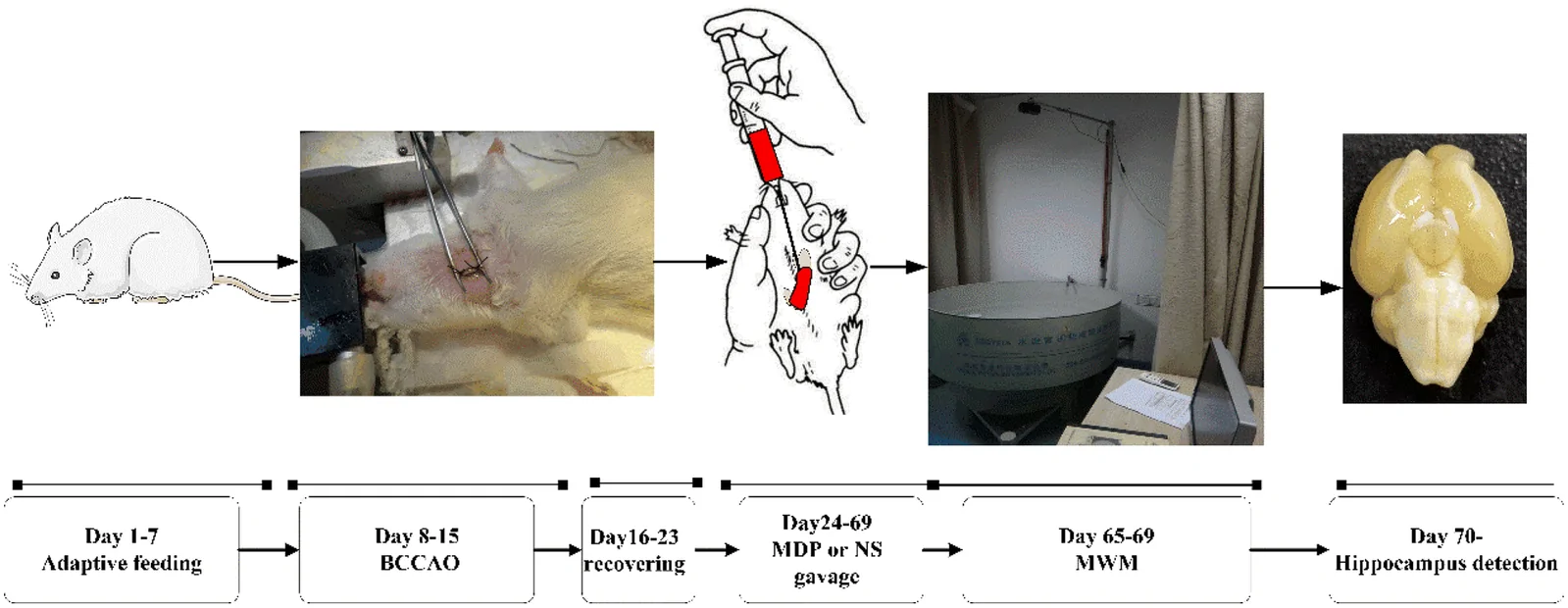
Schematic diagram of the experimental workflow.

#### 2.2.2 Preparation of MDP concentrated decoction

MDP concentrated decoction is prepared and provided by Hubei Hospital of Traditional Chinese Medicine (lot number: Z20150027). The prescription is composed of 14 kinds of Chinese herbs including: Dioscorea,30 g; Prepared Rehmannia,24 g; Processed Polygonum Multiflorum,24 g; Codonopsis,20 g; White Peony,20 g; Codonopsis Pilosula,20 g; Cinnabar, 12 g; Angelica Sinensis, 20 g; Acorus Tataricus,10 g; Astragalus,18 g; Poria,18 g; Eucommia,12 g; Goji Berries,18 g and Schisandra,10 g. All herbs were blended together and 400 mL water was added, then they were boiled, concentrated, filtrated and refined, they were condensed into 250 mL decoction(about 1 mL decoction contains about 1 g complex extract from these raw herbs) and preserved in bottle in refrigerator at 4℃ for use, the free radical scavenging activity can last for 90 days at 4℃ (15). All herbs were collected from the same batch to ensure consistent drug concentrations, and the expiration date of the drug is up to 6 months.

#### 2.2.3 Morris water maze test

The Morris water maze (MWM) test was performed using the MT-200 video tracking analysis system (Chengdu TaiMeng Technology Co., Ltd.). The MWM devices consiste of a white painted circular pool (120 cm in diameter and 50 cm in depth) in which the rats were trained to escape in the water by swimming to a hidden platform (11 cm in diameter) 1.5 cm beneath the surface, the location of which was only hinted by the curtain surrounded the pool. The rats behaviors are monitored and recorded by a video camera which is suspending over the pool and connected to a computer. The water was maintained at approximately 22 °C and made opaque with milk (about 500 g) throughout the training and testing period. The rats’ heads were stained yellow by tannic acid so that they could be clearly captured by the video tracking system. The pool was divided into four quadrants by compute: I(Target), II (Adjacent to I), III(Opposite to I) and IV(Between I and III). The computer has a computerized tracking system (Chengdu Taimeng Science and Technology Co., Ltd. Chengdu, China) to deal with the reference and compute the values such as escaping distance and escaping latency and so on.

The Morris water maze test was conducted in successive four days including escape test and probe test. The first 3 days were the training phase. 2 training trials per day for 3 consecutive days with an inter-trial interval of 30–40 min. At the beginning of each trial, the rats were placed into one of the four quadrants facing the wall. Each rat was allowed 120 s to locate and mount the platform; 30 s after the rat mounted the platform, it was rescued and moved to a drying cage and warmed with a heat lamp. The rats that failed to locate the platform within 120 s were gently guided to the platform and required to remain there for 60 s to remember the site by surrounding environment prior to being transferred to the drying cage. The amount of time spent locating and mounting the platform (escaping latency) and the swimming pathway (escaping distance) were calculated from the recorded videos using Morris water maze software. On the fourth day, a probe test was performed in which the platform was removed. The rats were placed into the water in quadrants III and were allowed to swim freely for 120 s The frequency of crossing the platform and the duration and distance in the target quadrant were recorded.

#### 2.2.4 Tissue collection, hippocampal section preparation, and HE staining

After the intervention, rats were deep anesthetized with 4% isoflurane inhalation. Then rats were thoracotomized, and the hearts were exposed, and they were perfused with normal saline from the apex of the hearts until their livers turned pale. Brains were removed, fixed in 4% paraformaldehyde, and stored at −20 °C. The hippocampus was dissected, dehydrated in ethanol, cleared in xylene, embedded in paraffin, and in the end they were cut into 30*μ*m sections.

Sections were deparaffinized, stained with hematoxylin and eosin (HE), and examined under 100× and 400× optical microscopes to evaluate histopathological features in the hippocampal CA1 region.

Notably, the same animals could provide tissue for multiple examinations where tissue availability permitted (e.g., the hippocampus of one hemisphere for Western blotting and the contralateral hippocampus for histological analyses), so that the total number of animals used across all assays was consistent with the final group sizes (*n* = 9, 12, and 13 for Sham, Model, and MDP groups, respectively).

#### 2.2.5 Immunohistochemistry and microvessel density (MVD) analysis

According to the results of MWM, the nine rats with the best behavioral performance of each group were selected for the immunohistochemical analyses of Bax, Bcl-2, NF-*κ*B p65, MAP-2, synaptophysin, PSD-95, Olig-2, and GFAP. For CD34 immunostaining and microvessel density (MVD) analysis, the experiments were re-performed using three animals per group. Paraffin sections of the hippocampus were subjected to antigen retrieval, endogenous peroxidase blocking, and primary antibody incubation with different dilution ratio, including rabbit anti-Bax (producer: abcam, batch number: ab32503, dilution: 1:200)：rabbit anti-Bcl-2 (producer: Wuhan Sanying Biotech. co, batch number: 12789-1-AP, dilution: 1: 100)：rabbit anti-NF-kB p65（producer: Wuhan Sanying Biotech. co, batch number: 10745-1-AP, dilution: 1: 100）: rabbit anti-MAP-2（producer: Wuhan Sanying Biotech. co, batch number: 17490-1-AP, dilution: 1: 50）：rabbit anti-Synaptophysin（producer: Wuhan Sanying Biotech. co, batch number: 17785-1-AP, dilution: 1: 120）; rabbit anti-PSD-95（producer: Wuhan Sanying Biotech. co, batch number: 20665-1-AP, dilution: 1: 30）: rabbit anti-Olig-2（producer: Wuhan Sanying Biotech. co, batch number:13999-1-AP, dilution: 1: 50）: rabbit anti-GFAP（producer: Santa cruz, batch number: sc-58766, dilution: 1: 150）; rabbit anti-CD34（producer: abcam, batch number: ab81289, dilution: 1:200, followed by HRP-conjugated secondary antibodies and DAB staining. Images were captured at 100× and 400× magnifications, and quantitative analysis was performed using IPP6.0 software on three 400× fields per section. Mean optical density (MOD), defined as the integrated optical density (IOD) divided by the measured area (i.e., the average staining intensity per unit area), was calculated and used for statistical comparison. For the re-performed CD34 analysis, four sections were prepared from each of the three animals per group and the mean optical density was measured in each section, yielding 12 values per group (3 animals  ×  4 sections).

For MVD analysis, following the Weidner method (16), individual endothelial cells or cell clusters stained by CD34 antibodies, with clear boundaries from surrounding microvessels and connective tissue, were counted as microvessels, excluding those with smooth muscle walls and a lumen diameter exceeding 8 red blood cells diameters. The MVD experiment was re-performed using three animals per group: three sections were prepared from each animal and three 400 ×   fields were examined in each section, yielding 27 microvessel density values per group (3 animals  ×   3 sections  ×  3 fields).

#### 2.2.6 Western blot analysis

Hippocampal tissue (0.1 g) was homogenized in RIPA lysis buffer, centrifuged to collect total protein, and protein concentration was determined using the BCA method. Equal amounts of protein were loaded, separated by denaturing polyacrylamide gel electrophoresis and transferred to polyvinylidene difluoride membrane. Membranes were blocked with 8% skim milk, incubated with primary antibodies including rabbit anti-GAPDH （37KD，producer: Hangzhou Xianzhi Biotech Co.，batch number: AB-P-R 001)；rabbit anti-Ang-1（70KD，producer: Wuhan Sanying Biotech. co, batch number: 23302-1-AP), rabbit anti-Ang-2（75KD，producer: abcam, batch number: A0698), rabbit anti-tie2 (126KD，producer: abcam, batch number: A7222)，rabbit anti-VEGF（45KD，producer: Wuhan Sanying Biotech. co, batch number: 19003-1-AP) overnight at 4 °C, and then incubated with HRP-conjugated secondary antibodies （producer: Wuhan Boster Biotech Co.，batch number: BA1054. At last, bands were visualized using ECL reagents, and protein expression levels were quantified by analyzing the gray values of the bands with IPP6.0 software, normalized to GAPDH. **Three animals per group were used for Western blot analysis.**

#### 2.2.7 Transmission electron microscope (TEM) examination

Two rats from each group were examined by TEM examination for the observation of nuclei and synapses to depict the neuronal death and the damage of synapses. Take 3 blocks of the hippocampus tissue which size was about 1 mm^3^ from each rat. By fixation, rinsing, dehydration, infiltration, embedding to make ultra-thin sections which thickness was about 60–80 nm. Then they were double stained with uranium and lead prior to be dried over a night. Select some representative sections to observe by TEM which were magnified to 4000 times.

#### 2.2.8 Statistical analysis

Measurement data are presented as mean ± standard deviation (mean ± SD) and were analyzed using GraphPad Prism 8.0. For the Morris water maze acquisition phase, escape latency and swimming distance measured across the training days were analyzed using two-way repeated-measures ANOVA with group (Sham, Model, and MDP) and training day as factors, followed by Bonferroni *post hoc* tests for between-group comparisons at each time point. Probe-test parameters and all other single-time-point measurements were analyzed by one-way ANOVA, starting with Levene's test for homogeneity of variances; Bonferroni *post hoc* tests were used when variances were equal and Tamhane's T2 when they were not. Pearson correlation analysis was performed between each pair of immunohistochemistry results using the animal-level mean values. In all analyses, the animal was treated as the experimental unit, and *P* < 0.05 was considered statistically significant.

## 3 Results

### 3.1 MDP improves the learning and memory abilities of CCH rats

In the four-day navigation test, all groups showed a decreasing trend in escape latency and escape distance. No significant between-group differences were found on the first two days. From the third day onward, the sham group exhibited significantly decreased escape distance and latency compared with the model group. On the fourth day, the MDP group showed a rapid decline in escape latency and distance, approaching the sham group, and both the sham and MDP groups demonstrated significantly decreased escape latency and distance compared with the model group (escape latency: 11.2 ± 4.7 s and 10.5 ± 2.4 s vs. 35.4 ± 22.1 s, *P* = 0.015; escape distance: 166.9 ± 57.2 cm and 172.7 ± 42.5 cm vs. 575.5 ± 367.0 cm, *P* = 0.043).

In the probe test, the sham and MDP groups showed significantly more platform crossings than the model group (11.6 ± 1.7 and 9.4 ± 1.5 vs. 3.7 ± 0.8). In addition, the sham and MDP groups spent significantly more time and swam significantly longer distances in the target quadrant than the model group (16.7 ± 4.3 s and 14.3 ± 2.8 s vs. 6.2 ± 1.8 s; 102.3 ± 24.5 cm and 98.8 ± 21.4 cm vs. 42.7 ± 11.3 cm). No significant difference was found between the sham and MDP groups in swimming distance in the target quadrant, except for platform-crossing frequency and time in the target quadrant. Total swimming distance did not differ significantly among the three groups; moreover, because the total swimming time was 120 s, swimming speed did not differ significantly among the three groups (Figure 2).

**Figure 2:**
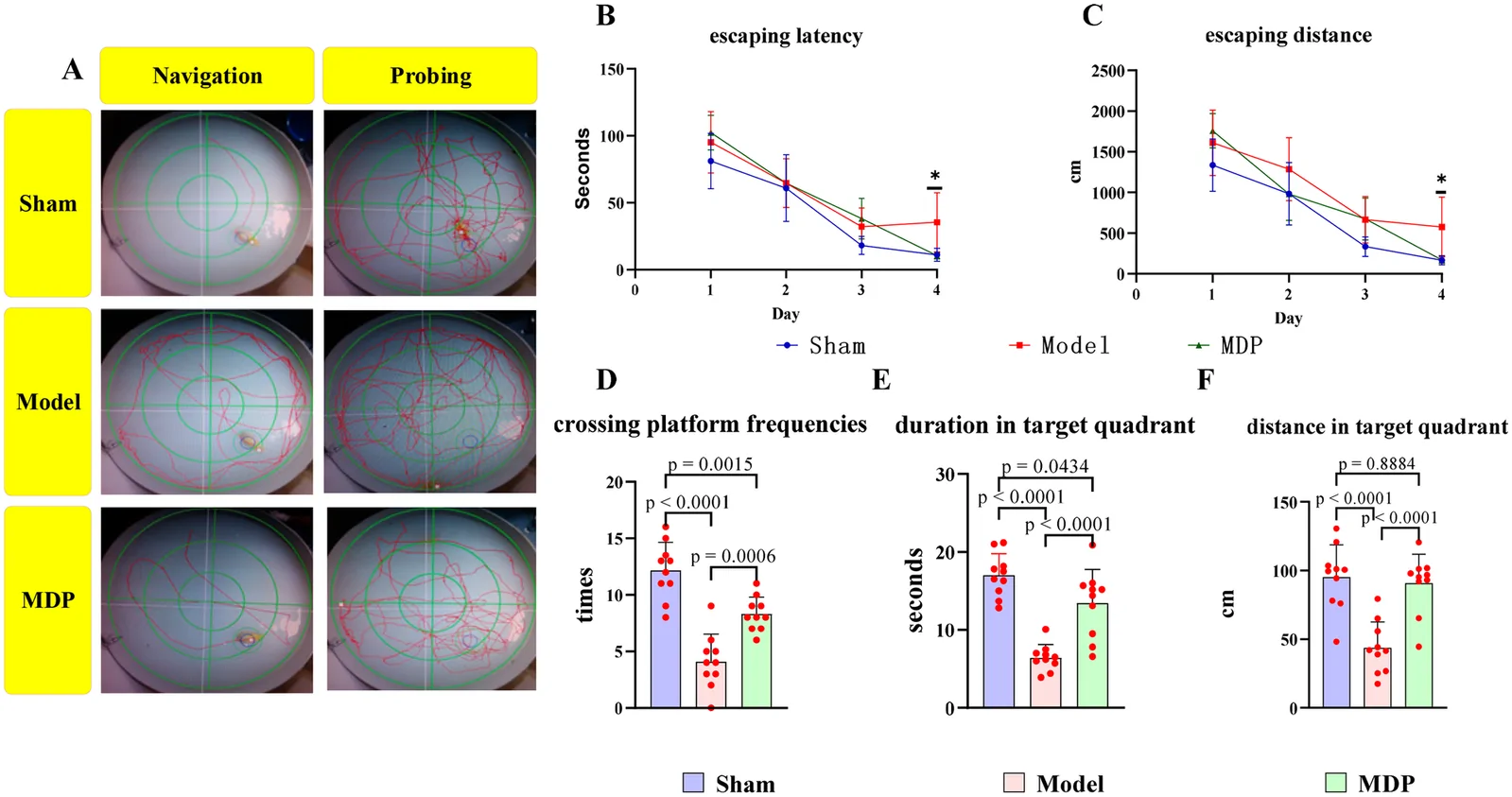
Behavioral results of the Morris water maze test. **(A)** Representative swimming trajectories of rats in navigation and probe tests. **(B,C)** Dynamic changes of escape latency and swimming distance during the 4-day navigation training. **(D–F)** Statistical analysis of platform crossing frequency, swimming time and swimming distance in the target quadrant during the probe test. Data are presented as mean ± SD (*n* = 9, 12, and 13 for Sham, Model, and MDP groups, respectively per group).

### 3.2 MDP alleviates hippocampal CA1 region pathological and ultrastructural damage in CCH rats

HE staining revealed that compared to the sham group, the model group had fewer and disorganized neurons in the CA1 region, with nuclear pyknosis and a looser radiate layer. In contrast, the MDP group showed a marked increase in neurons with more regular and compact structures.

Electron microscopy showed that in the model group, the presynaptic and postsynaptic membranes fused, synaptic vesicles seemed obscure, nuclear chromatin aggregated and moved to the nuclear membrane, and the nuclear membrane ruptured. However, the MDP group showed significant improvements compared to the model group. Higher-magnification insets further illustrated the clarity of synaptic vesicles and the integrity of pre- and postsynaptic membranes in the MDP group, supporting synaptic rescue (Figure 3).

**Figure 3:**
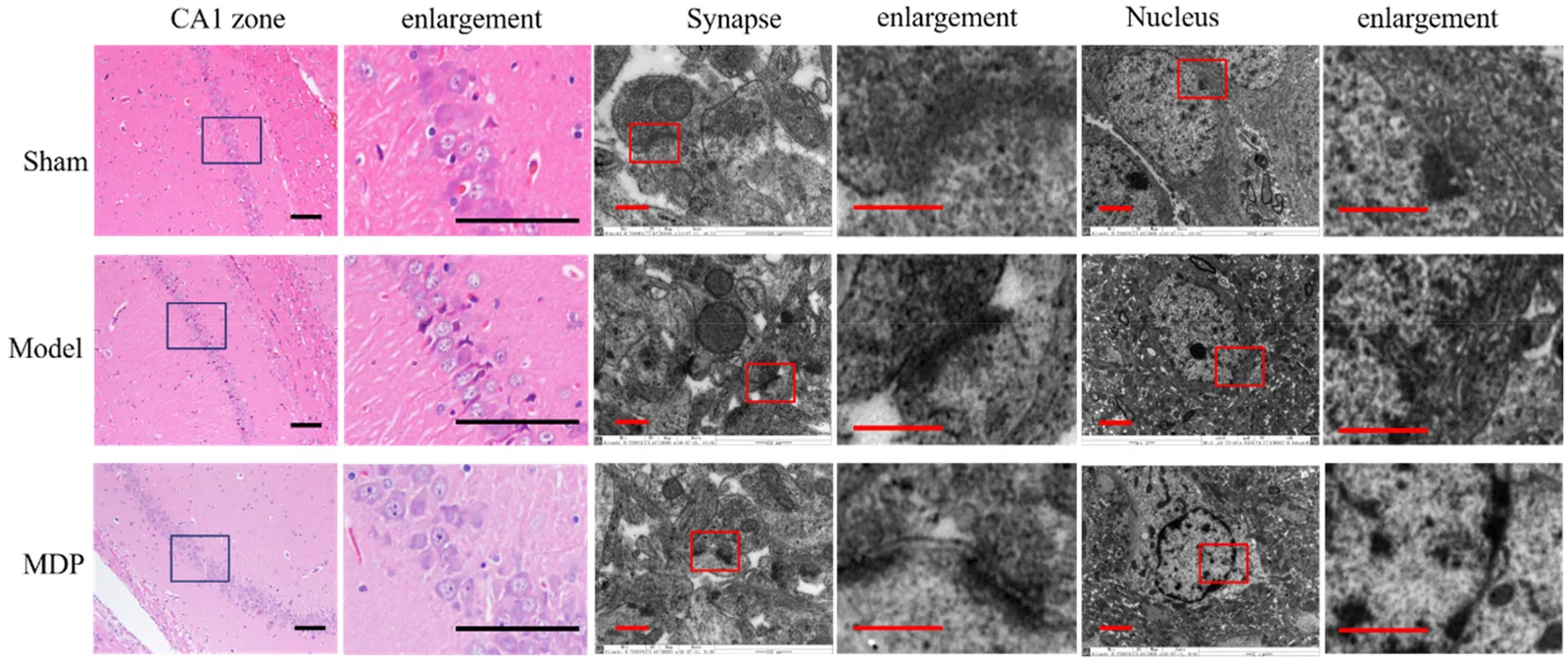
Representative images of histopathological and ultrastructural changes in the hippocampal CA1 region. Upper: HE staining of hippocampal CA1 neurons; Lower: TEM images of neuronal nuclei and synaptic ultrastructure. Scale bars: HE staining = 100 μm; TEM = 2 μm. Higher-magnification insets highlight synaptic ultrastructure, including synaptic vesicle clarity and pre-/postsynaptic membrane integrity.

### 3.3 MDP modulates inflammation and apoptosis-associated signaling in the hippocampus of CCH rats

Compared to the Sham group, the expression of Bax and NF-*κ*B p65 was significantly upregulated in the model group (0.0365 ± 0.00191 vs. 0.0085 ± 0.00062; 0.0161 ± 0.00090 vs. 0.0103 ± 0.00099), while Bcl-2 expression was significantly downregulated (0.0072 ± 0.00036 vs. 0.0100 ± 0.00046). MDP treatment evidently reversed these changes, significantly decreasing the expression of Bax and NF-*κ*B p65 (0.0202 ± 0.00084 vs. 0.0365 ± 0.00191; 0.0129 ± 0.00051 vs. 0.0161 ± 0.00090) and increasing the expression of Bcl-2 (0.0132 ± 0.00045 vs. 0.0072 ± 0.00036), consistent with attenuation of neuroinflammation and apoptosis-associated signaling (Figure 4).

**Figure 4:**
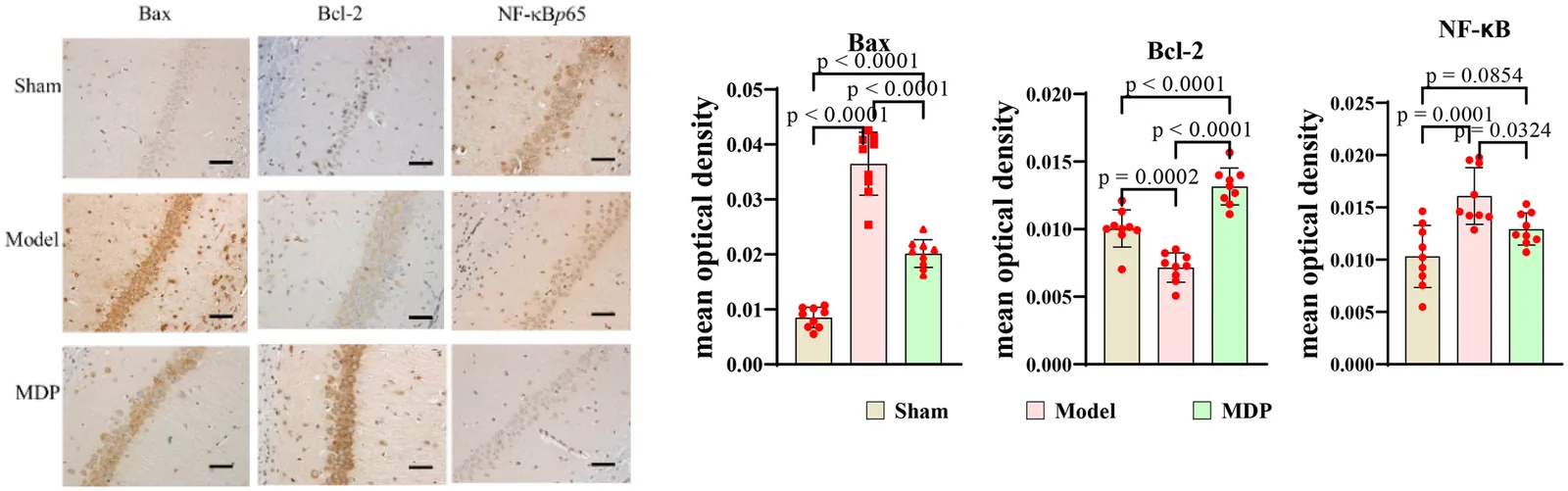
Expression of Bax, Bcl-2 and NF-*κ*B p65 in rat hippocampus detected by immunohistochemistry. Representative immunohistochemical images and quantitative analysis of mean optical density. Scale bar = 100 μm. Data are presented as mean ± SD (*n* = 9 in each group).

### 3.4 MDP promotes hippocampal neuronal and synaptic regeneration in CCH rats

In the model group, the expressions of MAP-2, SYP, and PSD-95 in the hippocampus were significantly downregulated compared to the Sham group (0.0099 ± 0.00034 vs. 0.0124 ± 0.00064; 0.0265 ± 0.00033 vs. 0.0359 ± 0.00092; 0.0228 ± 0.00032 vs. 0.0272 ± 0.00106). MDP treatment reversed these changes, the expressions of these three proteins were significantly higher than those in the CCH group (0.0157 ± 0.00047 vs. 0.0099 ± 0.00034; 0.0410 ± 0.00082 vs. 0.0265 ± 0.00033; 0.0359 ± 0.00105 vs. 0.0228 ± 0.00032), indicating its neuroprotective and synaptic regenerative effects (Figure 5).

**Figure 5:**
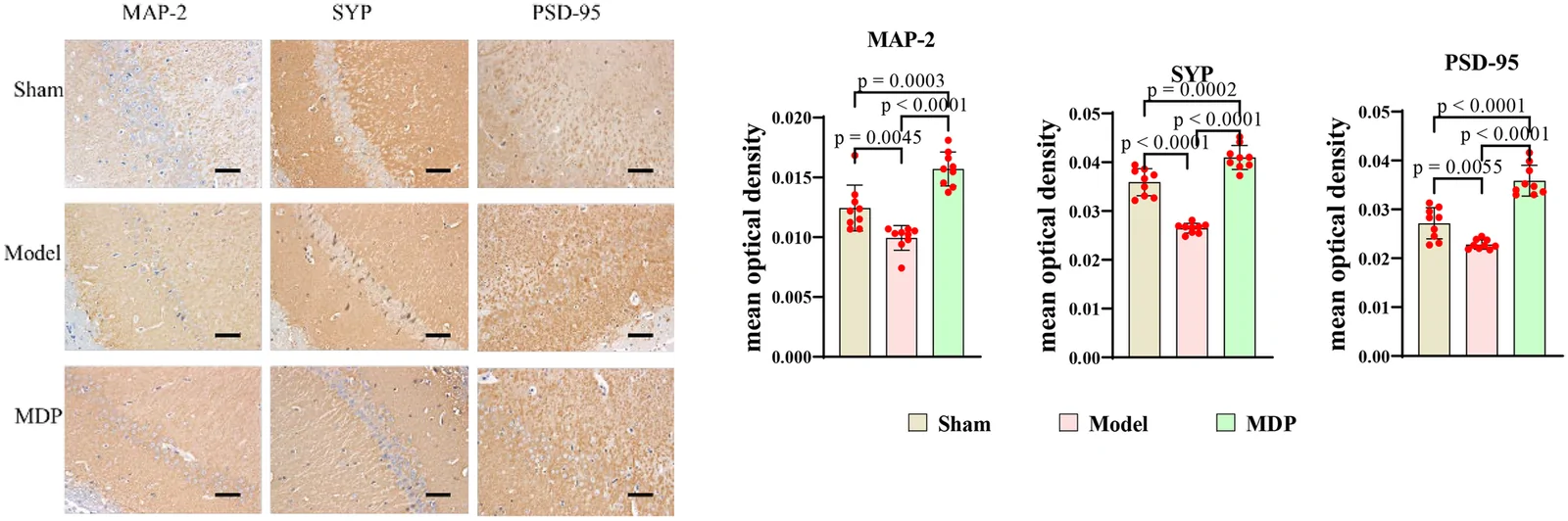
Immunohistochemical detection of MAP-2, synaptophysin (SYP) and PSD-95 in the hippocampus. Representative images and quantitative results of mean optical density. Scale ba*r* = 100 μm. Data are presented as mean ± SD (n = 9 in each group).

### 3.5 MDP inhibits hippocampal gliosis and restores oligodendrocyte-lineage integrity in CCH rats

Compared to the Sham group, GFAP expression in the hippocampus was significantly upregulated in the model group (0.0262 ± 0.00064 vs. 0.0101 ± 0.00046), while Olig-2 expression was significantly downregulated (0.0079 ± 0.00033 vs. 0.0126 ± 0.00038). MDP treatment significantly reduced GFAP expression and increased Olig-2 expression (0.0212 ± 0.00055 vs. 0.0262 ± 0.00064; 0.0112 ± 0.00069 vs. 0.0079 ± 0.00033), indicating its ability to inhibit gliosis and restore oligodendrocyte-lineage integrity. Because Olig-2 expression alone is not a direct measure of myelin integrity or remyelination, these findings are consistent with (although not definitive proof of) reduced demyelination and improved remyelination; confirmation with myelin-specific markers (e.g., MBP, PLP1, or Luxol fast blue staining) is warranted (Figure 6).

**Figure 6:**
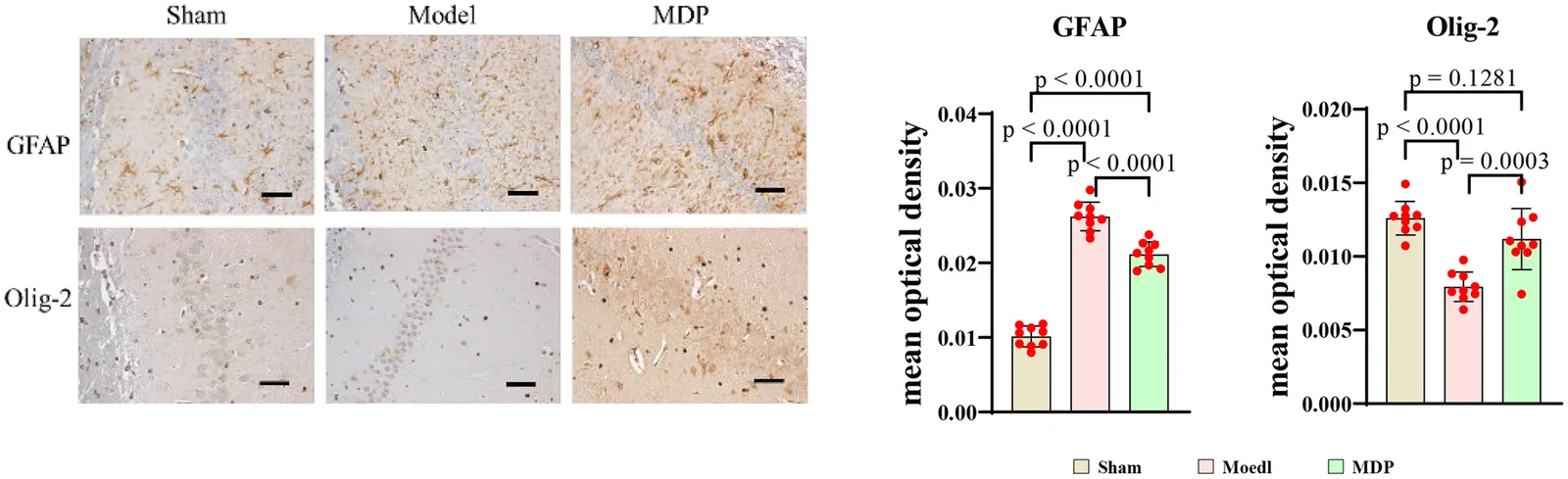
Immunohistochemical expression of GFAP and Olig-2 in the hippocampus. Representative staining images and statistical analysis. Scale bar = 100 μm. Data are presented as mean ± SD (n = 9 in each group).

### 3.6 MDP regulates the expression of ang/Tie2/VEGF pathway proteins in the hippocampus of CCH rats

In the model group, the expression of Ang-1, Ang-2, the ratio of Ang-1/ Ang-2,Tie-2, and VEGF in the hippocampus was significantly upregulated compared to the Sham group (0.597 ± 0.090 vs. 0.345 ± 0.058; 0.592 ± 0.035 vs. 0.442 ± 0.068; 1.007 ± 0.113 vs. 0.779 ± 0.046; 0.703 ± 0.025 vs. 0.524 ± 0.044; 0.706 ± 0.075 vs. 0.486 ± 0.030). MDP treatment further and significantly increased the expression of Ang-1, Ang-2, Tie-2, and VEGF (reaching 0.750 ± 0.060, 0.771 ± 0.017, 0.909 ± 0.019, and 0.844 ± 0.034, respectively; all *P* < 0.01 when compared with the CCH group) and decreased the Ang-1/Ang-2 ratio (0.972 ± 0.077 vs. 1.007 ± 0.113). The Ang-1/Ang-2 ratio was 0.779 ± 0.046, 1.007 ± 0.113, and 0.972 ± 0.077 in the Sham, Model, and MDP groups, respectively (Figure 7). These results suggest that MDP stabilizes vascular structure and supports vascular remodeling, thereby improving the hippocampal microvascular network.

**Figure 7:**
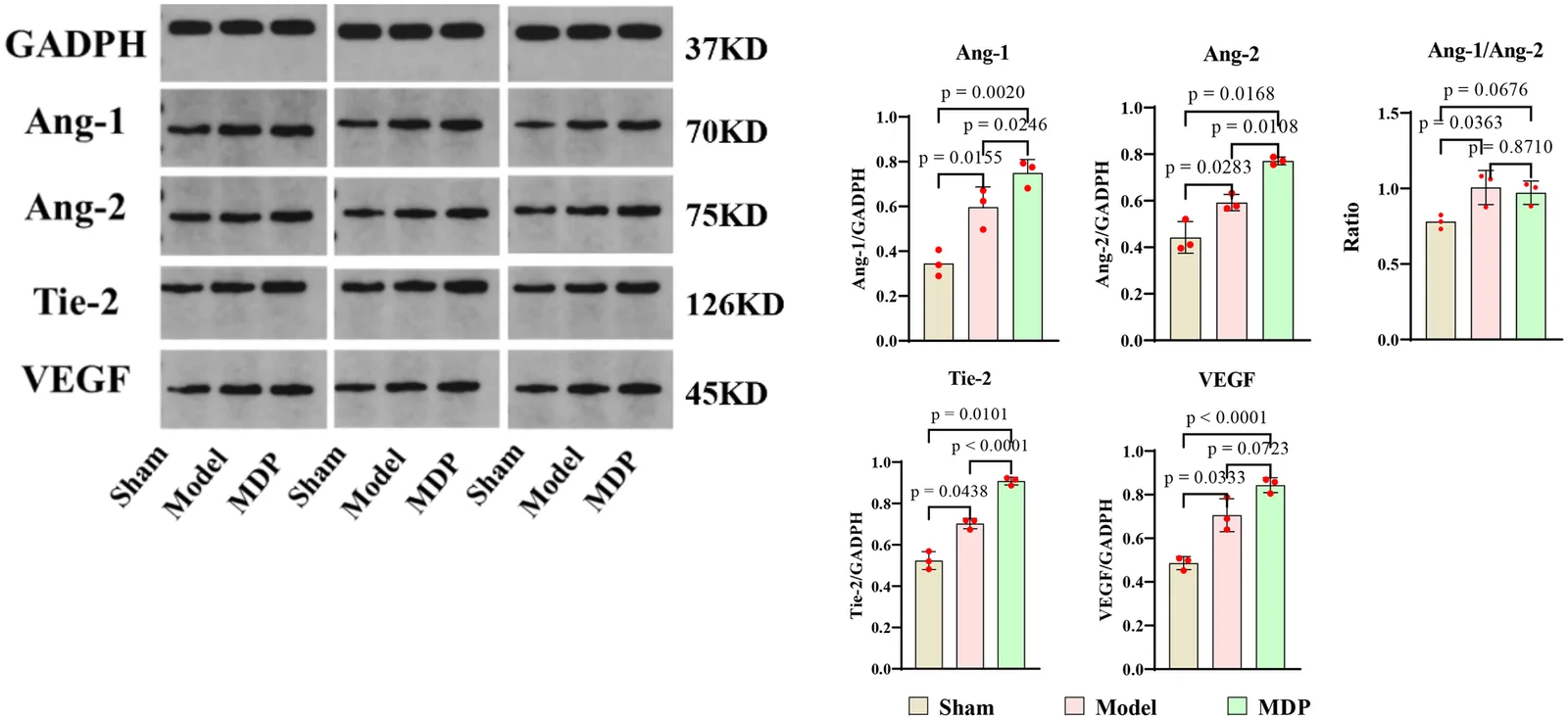
Western blot analysis of Ang-1, Ang-2, the ratio of ang-1/ang-2, Tie2 and VEGF expression in the hippocampus. Representative blot bands and relative quantitative results normalized to GAPDH. Data are presented as mean ± SD (n = 3 animals per group for Western blot analysis).

### 3.7 MDP promotes vascular stabilization and remodeling of the hippocampal microvasculature in CCH rats

In the model group, CD34 expression in the hippocampus was significantly upregulated and microvessel density (MVD) was significantly increased compared with the Sham group (0.00341 ± 0.00089 vs. 0.00082 ± 0.00036; 21.19 ± 2.62 vs. 5.78 ± 1.65). MDP treatment significantly reduced CD34 expression and MVD in the model rats (0.00208 ± 0.00057 vs. 0.00341 ± 0.00089; 14.93 ± 1.92 vs. 21.19 ± 2.62). However, CD34 expression and MVD in the MDP group remained significantly higher than those in the Sham group. Because an increase in microvessel density alone does not demonstrate *de novo* functional angiogenesis, and CD34 is not a specific marker of newly formed functional vessels, the reduction of CD34 expression and MVD, together with the upregulation of Ang-1/2, Tie-2, and VEGF and a balanced Ang-1/Ang-2 ratio, more appropriately indicate that MDP promotes the stabilization and remodeling of the excessive immature compensatory microvasculature rather than further pathological angiogenesis (Figure 8).

**Figure 8:**
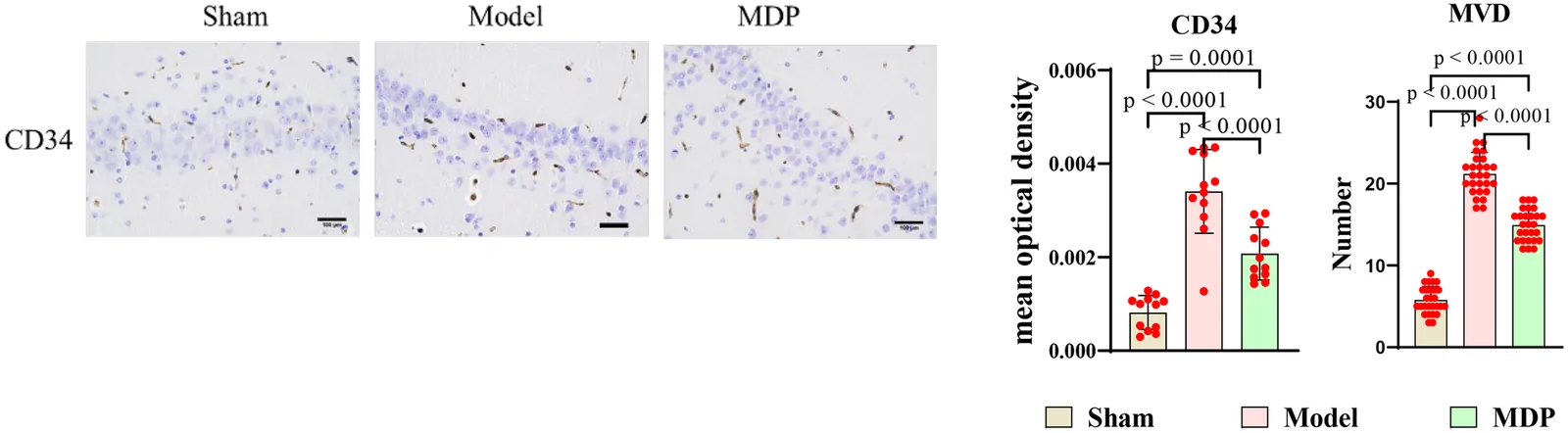
CD34 expression and microvessel density (MVD) in the hippocampus. Immunohistochemical images of CD34 and statistical analysis of CD34 mean optical density and MVD. Scale bar = 100 μm. Data are presented as mean ± SD (CD34: 12 values per group, obtained from three animals  ×  four sections; MVD: 27 values per group, obtained from three animals  ×  three sections  ×  three fields; see Section 2.2.5).

### 3.8 Coordinated changes in the components of the hippocampal NVU in CCH rats

Pearson correlation analysis revealed that NF-*κ*B expression was significantly positively correlated with Bax/Bcl-2 ratio and GFAP expression but negatively correlated with MAP-2, SYP, PSD-95, Olig-2, and CD34 expression. Additionally, MAP-2, SYP, PSD-95, and Olig-2 expression showed significant positive correlations with CD34 expression, and MAP-2 was also significantly positively correlated with SYP, PSD-95, and Olig-2 expression. These findings reveal significant statistical associations among key components of the NVU, including neurons, microvessels, synapses, and myelin, whereas inflammation-related markers were negatively associated with these components (Figure 9). It should be emphasized that correlation does not establish synergy or causality; these associations indicate coordinated changes rather than proven interactions among NVU components. Full Pearson correlation coefficients (r) and corresponding *P* values for all pairwise comparisons are provided in Figure 9.

**Figure 9:**
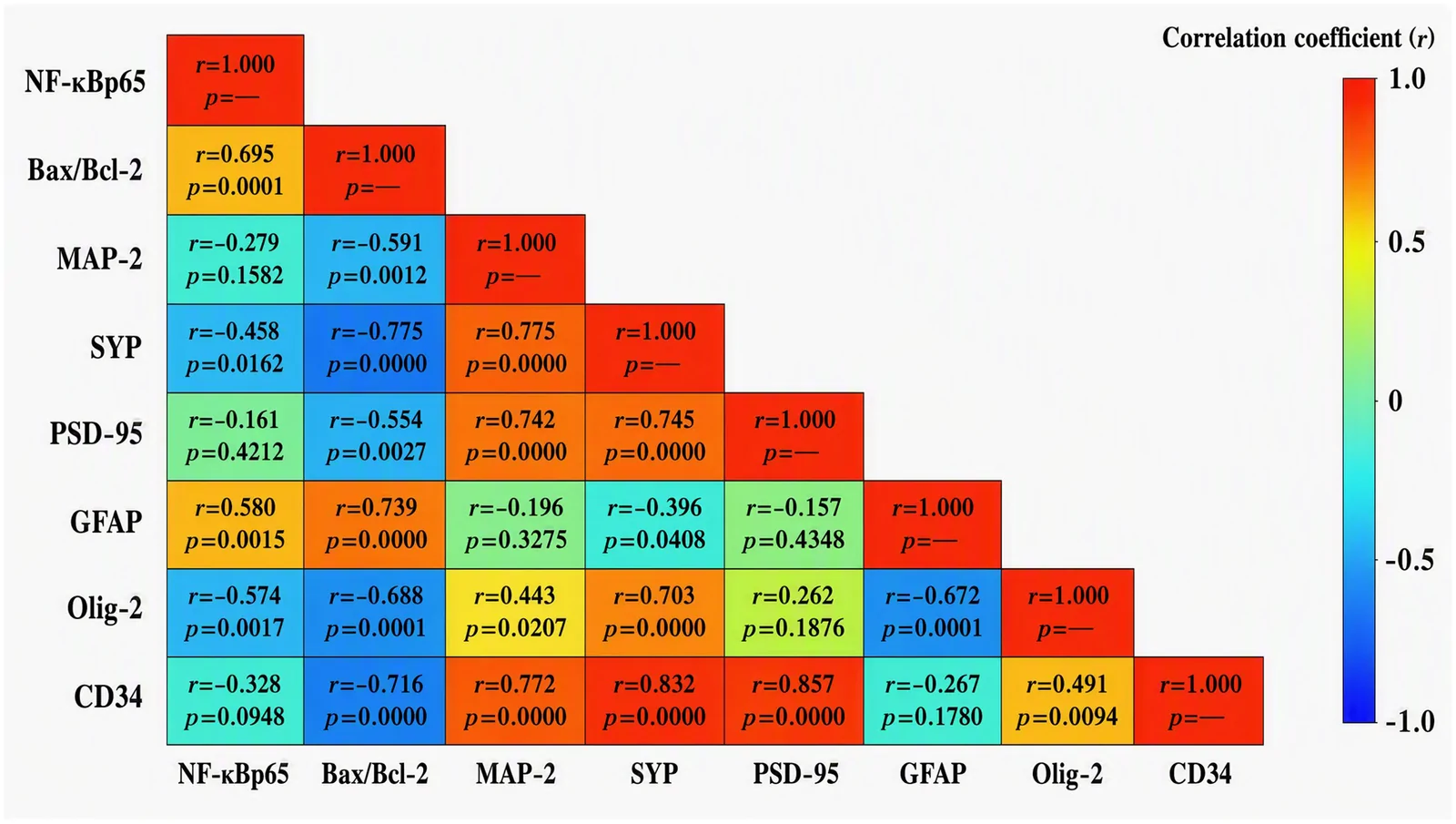
Pearson correlation analysis of NVU-related protein indicators, inflammatory and apoptotic markers. Correlation coefficients and statistical significance between all detected indexes are shown in the heatmap.

## Discussion

CCH triggers a series of neuropathological changes, including neuroinflammation, oxidative stress, and apoptosis, which are the core mechanisms underlying CCH-induced cognitive impairment (17). In its advanced stages, CCH further induces neurodegenerative changes, including damage to neurons, myelin, synapses, and glial cells, as well as abnormal glial activation and aberrant microvascular remodeling, which ultimately lead to irreversible neural circuit damage and due to the complex and multi-target pathological characteristics of CCH, multi-target and long-term comprehensive therapeutic strategies are required for its treatment.

Traditional Chinese medicine (TCM) prescriptions contain numerous active constituents that exert multi-target effects on cerebrovascular and neurodegenerative diseases. The main active substances of MDP include yam polysaccharide, Polygonum multiflorum polysaccharide, Rehmannia glutinosa polysaccharide, Lycium barbarum polysaccharide, diosgenin, *β*-asarone, and ferulic acid, among others, and these components have been reported to exert antioxidant, free radical-scavenging, anti-aging, anti-apoptotic, and neuroregenerative effects (18–21). In particular, diosgenin, the principal steroidal sapogenin of Dioscorea, promotes hippocampal axonal growth and restores memory function in a mouse model of Alzheimer's disease (17); *β*-asarone, the main active constituent of Acorus gramineus, ameliorates cognitive impairment and alleviates autophagy in mice with vascular dementia via the cAMP/PKA/CREB pathway (18); ferulic acid, a phenolic acid enriched in Angelica sinensis, consistently improves cognitive function in animal models of dementia (19); and yam polysaccharides, the principal bioactive fraction of Dioscorea opposita, exhibit broad immunomodulatory and antioxidant activities (20). Our group has previously demonstrated that MDP promotes neuroplasticity and vascular remodeling in CCH (12, 13), and the present study extends these findings by systematically characterizing the coordinated changes of the hippocampal NVU components in CCH. These pharmacological activities are highly consistent with the neuroprotective and NVU-restoring effects of MDP observed in the present study. The neurovascular unit (NVU) is a functional complex composed of neurons, glial cells, vascular endothelial cells, pericytes, and the extracellular matrix, emphasizes the close correlations between brain cells and microvessels in development, structure and function, as well as their coordinated response to injury (22). In this study, CCH led to a significant decline in rats’ learning and memory abilities. In the MWM test, compared to the sham group, the model group showed severe learning and memory impairments in navigation and probing test. MDP significantly improved the behavioral performance of model rats. In this study, we explored its specific mechanisms by investigating its effects on hippocampal neuroinflammation, neuronal apoptosis, synaptic function, glial remodeling, and angiogenesis in the CCH rats.

Diosgenin, *β*-asarone, ferulic acid, and yam polysaccharides are candidate bioactive molecules of MDP that may act on the hippocampal NVU. Given the multi-herbal nature of the MDP formulation, future chemical fingerprinting combined with multi-component quantification is warranted to ensure batch-to-batch consistency and bioactivity (23–25). Our findings also align with recent clinical and translational evidence demonstrating that carotid revascularization (carotid endarterectomy, CEA) improves cognitive function and restores cerebral blood flow and white matter integrity in patients with carotid artery stenosis-associated CCH (6), supporting the restoration of the hippocampal NVU as a key therapeutic strategy for CCH.

Compared to the cerebral cortex, the hippocampus contains higher levels of inflammatory response and cell death related genes such as *Serinc3, Lcn2, Pmai1, Ackr1,* and *Plat,* making it more vulnerable to neuroinflammation (26, 27). Ischemic damage can activate TLR4 and the NLRP3 inflammasome via damage associated molecular patterns (DAMPs) (4, 28), triggering an inflammatory cascade and exacerbating neuronal damage and cognitive dysfunction. In this study, the model group showed significantly increased expression of NF-*κ*Bp65 and Bax, and decreased expression of MAP-2 and Bcl-2. NF-*κ*B is a key transcription factor that mediates multiple inflammatory signaling pathways its activation leads to the upregulation of pro-inflammatory cytokines such as TNF-α, IL-1β, and IL-6 (29, 30), which in turn aggravate neuroinflammation. Upregulated expression of BAX means more necroptosis, pyroptosis, or autophagy in hippocampus (31), and the increasing Bax/Bcl-2 ratio suggests marked cell apoptosis in CCH (32, 33). MAP-2 is a marker protein of mature neurons which regulates neurite outgrowth, neural development, and repairmen (34). These findings are consistent with CCH-induced activation of neuroinflammation and apoptosis-associated signaling in hippocampal neurons, which was also depicted by HE staining and electron microscopy. MDP significantly reduced these NF-*κ*B p65- and Bax/Bcl-2-related signals, suggesting anti-inflammatory and neuroprotective effects; direct evidence of neuroinflammation and neuronal apoptosis (e.g., microglial activation markers such as Iba1/CD68, or co-labeling of the neuronal marker NeuN with cleaved caspase-3 or TUNEL staining) would further substantiate these conclusions. As a long term effect, sustaining neuroinflammation continuously activates microglia, affecting neural circuit development and leading to neurodegeneration (35), which may contribute to the irreversible cognitive dysfunction.

Microglia are also involved in CCH-induced white-matter demyelination and white-matter lesions (WMLs), which are main drivers of vascular cognitive impairment (VCI) and dementia (36, 37). In addition, CCH activates astrocytes, further promoting neuroinflammation and gliosis. In this study, the model group showed obviously increased GFAP expression, indicating astrocyte activation, and decreased Olig-2 expression, suggesting impaired oligodendrocyte-lineage integrity, which is compatible with (although not a direct measure of) demyelination. These findings were corroborated by HE staining, which revealed increased gaps in the radiation layer, pointing to WMLs and demyelination. These changes cause signaling and neural circuitry disruption, worsening cognitive function (38). MDP reversed these changes, suggesting that long-term MDP treatment can attenuate gliosis and support oligodendrocyte-lineage recovery. Because Olig-2 expression alone is not a direct measure of myelin integrity or remyelination, confirmation with myelin-specific markers (e.g., MBP, PLP1, MOG, MAG, or Luxol fast blue staining) is warranted to verify MDP-induced remyelination.

Synaptic plasticity is the cellular and molecular basis of learning and memory, and synaptic loss and dysfunction are important pathological features of CCH-induced cognitive impairment. Synaptophysin (SYP), located in the presynaptic membrane, is responsible for nerve growth and synaptic remodeling (39). PSD-95, located in the postsynaptic membrane, it is a Membrane-Associated Guanylate Kinase (MAGUK), which regulates synaptic plasticity and neurotransmission efficiency (40, 41). Their expression quantities reflect synapse quantities and function. In this study, CCH significantly reduced SYP and PSD - 95 expression, and electron microscopy showed synaptic structure damage, indicating synapse loss and dysfunction. MDP significantly increases the expression of these two proteins and improve synaptic structures, suggesting it protects against CCH induced synaptic damage and promotes synaptic regeneration. This is also one of the mechanisms by which MDP improves NVU structure and cognitive function.

The close structural and functional interaction between neurons and microvessels (i.e., neurovascular coupling) is a unique feature of the cerebral circulation and the basis of functional magnetic resonance imaging signals (42). Neurons can regulate nearby microvasculature to support brain metabolism and maintain homeostasis (43). Angiopoietins (Ang) are a family of vascular growth factors and ligands for TIE (Tyr kinase with Ig and epidermal growth factor homology domains 2) receptors, Ang/TIE axis regulates the stabilization and remodeling of the microvascular network to meet the needs of the environment (44). VEGF regulates endothelial cell proliferation, migration, survival, and vessel formation (45, 46). Under physiological conditions, Ang-1 dominates, binds to and phosphorylates the TIE2 receptor, ultimately promotes cell survival and vascular stability. Ang-2 is upregulated under pathological conditions such as hypoxia or inflammation, and acts as a context-dependent agonist/antagonist of the Ang-1/Tie2 axis, when Ang-1 is absent, Ang-2 acts as an agonist, but when Ang-1 is present, Ang-2 acts as a dose-dependent antagonist (47). During vascular remodeling and inflammation, Ang-2 expression is increased in endothelial cells, which destabilizes quiescent vessels, and promotes the vascular sprouting, angiogenic and migratory effects of VEGF (47). Accordingly, Ang-2 functions as a dynamic regulator of the Ang-Tie2 axis, acting as a built-in switch that controls the transition of the quiescent endothelium toward the activated, responsive endothelium; its net effect is context-dependent and governed by the local balance between Ang-1 and Ang-2.

CD34 is a vascular endothelial cell marker; when overexpressed, it indicates increased angiogenesis and MVD (48, 49). In this study, the model group showed significantly increased expression of Ang-1/2, Tie-2, and VEGF, along with elevated CD34 expression and MVD, indicating compensatory angiogenesis due to inflammatory and injury stimuli. MDP further boosted Ang-1/2, Tie-2, and VEGF expression, but reduced CD34 expression and MVD. This suggests that the significant increase in Ang-1/2 helps maintain vascular stability while reducing vascular damage and the formation of ineffective or malformed vessels. Importantly, Ang-2 can act as a context-dependent Tie2 antagonist in the presence of Ang-1 and as a partial agonist under conditions of Ang-1 scarcity (47), whereas VEGF exerts dual functions as both an angiogenic factor and a neurotrophic signal supporting neuronal survival and synaptic function. The balanced Ang-1/Ang-2 ratio, together with the coordinated upregulation of Ang-1/2, Tie-2, and VEGF observed in the MDP group, therefore indicates that MDP promotes adaptive vascular stabilization and remodeling rather than dysfunctional vascular sprouting, consistent with our group's previous findings (13). In addition, an increase in MVD does not by itself demonstrate *de novo* functional angiogenesis, and CD34 is not a specific marker of newly formed functional vessels; therefore, the expression of Ang-1/2, Tie-2, and CD34 more appropriately supports vascular remodeling and vascular stabilization rather than simple promotion of angiogenesis.

Notably, our correlation analysis revealed a high degree of coordination among the components of the hippocampal NVU in CCH rats: the formation and number of microvessels adapt to the number and function of neurons, synapses, and myelination, and there is a fine regulation mechanism between them. In addition, these modulatory proteins increased synchronously during damage, reflecting the body's compensatory needs. However, they were negatively correlated with inflammation and apoptosis. This indicates that the NVU behaves as a coordinated functional unit and that its components exhibit statistically associated changes in response to CCH-induced damage; however, these correlational findings do not establish synergy or causal interactions among the components. The specific coordinated mechanisms are complex, featuring diverse and bidirectional features of these regulatory proteins. For instance, VEGF not only promotes angiogenesis but also supports neuronal survival and synaptic function, which is consistent with the coordinated NVU-restoring effects of MDP observed in this study. The exact mechanisms require further exploration.

A key translational limitation of the present study is the discrepancy between the experimental model and the clinical condition of CCH. In humans, CCH is predominantly driven by atherosclerotic carotid artery stenosis in older adults, and the neuroinflammatory, oxidative stress, and blood–brain barrier phenotypes differ substantially between young, otherwise healthy BCCAO rats and aged, atherosclerotic patients (2, 50). Although the BCCAO model is widely used to recapitulate chronic cerebral hypoperfusion, it does not reproduce the atherosclerotic and age-related vascular pathology that underlies most human CCH. Accordingly, the boundaries for extrapolating our preclinical findings to human disease must be defined with caution, and future studies using aged or atherosclerosis-prone animal models, together with clinical validation, are warranted.

The study has other limitations. For example, differences in hippocampal structure and blood supply between rats and humans mean that animal experimental results may not fully reveal human CCH mechanisms. In addition, the chemical composition of the MDP decoction has not yet been fully characterized, and the blood–brain barrier permeability, brain concentrations, and the relative contributions of individual constituents to the observed NVU-restoring effects remain to be clarified in future pharmacokinetic and pharmacodynamic studies. Most importantly, we did not perform pharmacokinetic profiling of MDP's bioactive constituents in brain tissue, which would more directly link these constituents to their central pharmacological effects. Finally, the study did not deeply explore the specific mechanisms of each factor in CCH, and these require further research.

In addition, this study has several further limitations. First, only male rats were used, and potential sex-specific effects of MDP could not be evaluated. Second, direct measurements of cerebral perfusion and blood–brain barrier permeability were not performed, so the relationship between the observed molecular changes and actual cerebral blood flow remains indirect. Third, no mechanistic intervention (e.g., pathway inhibitors or genetic manipulation) was applied, and thus the causal roles of the Ang/Tie2/VEGF and inflammatory signaling pathways in mediating MDP's effects were not directly demonstrated. Fourth, a dose-response analysis was not performed, and the single-dose design limits the dose-basis evidence for clinical translation; future dose-gradient studies would provide a stronger theoretical basis for clinical transformation. Finally, the correlational design of this study cannot establish causal relationships among NVU components, and the observed changes should be interpreted as associative. These issues warrant further investigation in future studies.

## 4 Conclusion

This study provides experimental evidence for the clinical application of MDP in the treatment of CCH and vascular dementia, and highlights the hippocampal NVU as a potential therapeutic target for CCH-induced cognitive impairment.

## Data Availability

The raw data supporting the conclusions of this article will be made available by the authors, without undue reservation.
